# When inclusive leadership promotes innovation: the role of team psychological safety and leader power distance orientation

**DOI:** 10.3389/fpsyg.2026.1868768

**Published:** 2026-07-02

**Authors:** Aiqi Zhang, Juhee Hahn

**Affiliations:** 1 The Graduate School, Chung-Ang University, Seoul, Republic of Korea; 2 Department of Business Management, Chung-Ang University, Seoul, Republic of Korea

**Keywords:** employee innovative behavior, inclusive leadership, leader power distance orientation, multilevel model, team psychological safety

## Abstract

**Introduction:**

Inclusive leadership is important for employee innovation because it encourages employees to speak up, share ideas, and try new ways of working. However, inclusive leadership may not have the same effect in all teams. It is therefore necessary to explain how inclusive leadership promotes innovation and when its effect may become weaker. Based on social information processing theory, this study examines the mediating role of team psychological safety and the moderating role of leader power distance orientation.

**Methods:**

Data were collected in three waves from 475 employees nested within 93 teams led by 93 team leaders in Chinese IT firms. Multilevel analysis was used to test the proposed relationships.

**Results:**

Inclusive leadership was positively related to employee innovative behavior and team psychological safety. Team psychological safety further promoted employee innovative behavior. Leader power distance orientation weakened the positive relationship between inclusive leadership and team psychological safety. The positive effect of inclusive leadership on team psychological safety was significant when leader power distance orientation was low, but non-significant when it was high. The conditional indirect effect of inclusive leadership on employee innovative behavior through team psychological safety was also significant only when leader power distance orientation was low.

**Discussion:**

These findings show that inclusive leadership promotes employee innovation by creating a psychologically safe team environment, but this process becomes weaker when leaders have a stronger power distance orientation. This study helps explain why inclusive leadership may be more effective in some teams than in others.

## 1 Introduction

As China's information technology (IT) industry continues to grow rapidly, organizations face increasing pressure to innovate. Rapid technological change, intense competition, and high employee mobility make innovation essential for organizational competitiveness (Pea-Assounga and Bindel Sibassaha, 2024). In this context, employee innovative behavior has become an important source of organizational adaptation and long-term success (Liu et al., 2025). Therefore, understanding how leadership promotes employee innovation has become an important topic in organizational research.

Inclusive leadership has received increasing attention because it emphasizes openness, accessibility, and support for employee participation (Jiang et al., 2022). Inclusive leaders are willing to listen to different opinions, encourage employees to share ideas, and create opportunities for participation (Shore and Chung, 2022). Previous studies have shown that inclusive leadership is associated with positive employee outcomes, such as work engagement, affective organizational commitment, employee creativity, and innovative behavior (Choi et al., 2015; Fang et al., 2019). However, although this literature has provided valuable insights, two issues require further attention.

First, existing research has often explained the effect of inclusive leadership on innovation from the perspective of individual employee perceptions (Liu et al., 2026). Less is known about how inclusive leadership shapes a shared team climate, such as team psychological safety, which in turn influences employee innovative behavior (Li and Tang, 2022). This issue is important because leadership does not only affect individual attitudes. It also shapes shared interpretations of what behaviors are accepted and supported within teams (Liu et al., 2026). Team psychological safety, defined as a shared belief that the team is safe for interpersonal risk-taking (Patil et al., 2023), may be a key team-level mechanism. When team members feel psychologically safe, they are more willing to express ideas, ask questions, discuss problems, and try new approaches (Kumar, 2024). Therefore, examining team psychological safety can help explain how inclusive leadership promotes employee innovative behavior through a team-level psychological climate.

Second, the positive effect of inclusive leadership may not be equally strong in all teams. Inclusive leadership sends a signal that employees are allowed to speak up, participate, and take interpersonal risks (Jiang et al., 2022). However, this signal may be interpreted differently depending on other cues provided by the leader. Leader power distance orientation may influence whether employees perceive inclusive behaviors as sincere and reliable (Orekoya, 2024). Leaders with low power distance orientation tend to value equality, participation, and closer interaction with employees. Low power distance is related to smaller manager-employee distance, more consultation, and stronger employee participation in decision-making (Sagie and Aycan, 2003). In this case, inclusive behaviors are consistent with the leader's underlying values, and employees may perceive the leader's openness as credible.

In contrast, leaders with high power distance orientation tend to place greater emphasis on hierarchy, authority, and obedience to managerial decisions (Wang and Guan, 2018). Even when such leaders display inclusive behaviors, employees may receive mixed signals. On the one hand, the leader may appear open, accessible, and willing to listen (Carmeli et al., 2010). On the other hand, the leader's authority-oriented values may still communicate distance and hierarchy (Li and Tang, 2022). This inconsistency may make employees uncertain about whether it is truly safe to express different opinions, question existing practices, or challenge the leader's views (Lee and Dahinten, 2021). As a result, the positive effect of inclusive leadership on team psychological safety may become weaker when leader power distance orientation is higher.

Based on this reasoning, this study examines whether inclusive leadership promotes employee innovative behavior through team psychological safety and whether leader power distance orientation weakens the relationship between inclusive leadership and team psychological safety. Using data from Chinese IT firms, this study develops a multilevel model that links leadership behavior, team psychological climate, leader values, and employee innovative behavior.

This study offers three contributions to the inclusive leadership and employee innovation literature. First, it extends inclusive leadership research by examining team psychological safety as a team-level mechanism linking inclusive leadership to employee innovative behavior. This shifts the focus from individual employee perceptions to a shared team process. Second, it clarifies an important boundary condition of inclusive leadership by showing that leader power distance orientation weakens the positive effect of inclusive leadership on team psychological safety. This finding suggests that inclusive leadership may not work equally well in all teams. Third, this study integrates leader behavior and leader values. It shows that the effect of inclusive leadership depends not only on whether leaders behave in an open and supportive way, but also on whether their underlying values are consistent with these behaviors.

## 2 Theoretical background and hypotheses

Drawing on social information processing theory (Salancik and Pfeffer, 1978), this study argues that employees interpret leadership behaviors as important social cues about what is accepted, valued, and safe in the workplace. Inclusive leadership signals openness, accessibility, and support for employee participation. However, the meaning of this signal may depend on whether the leader's underlying values are consistent with inclusive behaviors. Inclusive leadership sends signals of openness, availability, and accessibility, which can promote psychological safety (Carmeli et al., 2010; Li and Tang, 2022). However, when leaders hold a high-power distance orientation, they may still emphasize hierarchy, authority, and obedience to managerial decisions (Dorfman and Howell, 1988; Wang and Guan, 2018). Therefore, this study examines team psychological safety as a team-level mechanism and leader power distance orientation as a boundary condition.

### 2.1 Inclusive leadership and employee innovative behavior

Employee innovative behavior refers to the generation, promotion, and implementation of new ideas at work (Handayani and Pendrian, 2023). Such behavior is important for organizational adaptation and competitiveness, but it also involves uncertainty and interpersonal risk. Employees who propose new ideas may face criticism, rejection, or negative evaluation, especially when their ideas challenge existing routines (Janssen, 2000).

Inclusive leadership can encourage employee innovative behavior by creating a supportive social environment. Inclusive leaders are open, accessible, and willing to listen to employees' ideas (Zhang and Zhao, 2024). By inviting employees to share views, encouraging participation, and responding constructively to suggestions, inclusive leaders communicate that employees' ideas are valued (Roberson and Perry, 2022). According to social information processing theory (Salancik and Pfeffer, 1978), employees use such leadership cues to judge whether innovative actions are expected and supported. When leaders show openness and respect, employees are more likely to believe that proposing new ideas is acceptable rather than risky.

Therefore, inclusive leadership may increase employees' confidence in expressing novel ideas, seeking new methods, and implementing innovative solutions. In this way, inclusive leadership provides a relational foundation that encourages employees to engage in innovative behavior.

H1: Inclusive leadership positively influences employee innovative behavior.

### 2.2 Inclusive leadership and team psychological safety

Team psychological safety refers to a shared belief among team members that the team is safe for interpersonal risk-taking (Sacramento et al., 2024). In psychologically safe teams, members feel able to express concerns, ask questions, admit mistakes, and propose different ideas without fear of embarrassment or punishment (Kumar, 2024). Because psychological safety reflects a shared team climate, it develops through repeated interactions and common interpretations of cues from leaders and other team members (Patil et al., 2023).

Inclusive leadership is likely to foster team psychological safety because inclusive leaders provide clear social cues that openness and participation are accepted in the team. Inclusive leaders are accessible, invite employees to express ideas, listen to different viewpoints, and show tolerance for mistakes (Woods et al., 2024). These behaviors reduce the interpersonal risk associated with speaking up. When employees observe that their leader responds constructively to suggestions and concerns, they are more likely to believe that expressing ideas is safe (Hubbart, 2024).

Over time, repeated inclusive behaviors can become shared signals within the team (Pless and Maak, 2004). Team members may collectively learn that questioning existing practices, discussing problems, and offering new ideas are acceptable behaviors. Therefore, inclusive leadership can shape not only individual perceptions but also a shared team climate of psychological safety.

H2: Inclusive leadership positively influences team psychological safety.

### 2.3 Team psychological safety and employee innovative behavior

Team psychological safety plays an important role in encouraging employee innovative behavior (Miao et al., 2020). Innovation requires employees to express new ideas, challenge existing practices, and try new solutions (Karimi et al., 2023). These actions are uncertain because new ideas may fail, be criticized, or disrupt established routines. Therefore, employees are more likely to engage in innovative behavior when they believe that the team environment is safe for interpersonal risk-taking.

Team psychological safety encourages innovation in several ways. First, when psychological safety is high, employees are more willing to voice novel ideas and share early-stage suggestions (Çetin, 2025). Second, psychological safety makes employees more willing to experiment and learn from mistakes, which is essential for innovation (Mehmood et al., 2022). Third, psychologically safe teams allow members to challenge existing practices and discuss alternative solutions without fearing negative interpersonal consequences (Weiss et al., 2023).

Thus, team psychological safety provides an important social foundation for employee innovation. When employees perceive that their team accepts open discussion, experimentation, and constructive disagreement, they are more likely to search for new methods, promote new ideas, and implement innovative solutions.

H3: Team psychological safety positively affects employee innovative behavior.

### 2.4 Moderating role of leader power distance orientation

Leader power distance orientation refers to the extent to which leaders accept and endorse unequal power distribution, hierarchy, and authority in leader-subordinate relationships (Zhang et al., 2025). Leaders with low power distance orientation are likely to place less emphasis on hierarchy and authority. They tend to value employee participation, equality, and closer interaction with employees (Sagie and Aycan, 2003). In contrast, leaders with high power distance orientation tend to emphasize authority, status differences, and obedience to leaders (Wang and Guan, 2018).

Although inclusive leadership sends a positive signal that employees are allowed to speak up and participate, this signal may be interpreted differently depending on the leader's power distance orientation (Guo et al., 2022). When leaders have low power distance orientation, their inclusive behaviors are more likely to be consistent with their underlying values. In this situation, employees are more likely to perceive the leader's openness as sincere and reliable. As a result, inclusive leadership can more effectively reduce interpersonal risk and strengthen team psychological safety.

However, when leaders have high power distance orientation, employees may receive mixed signals. On the one hand, the leader may show inclusive behaviors, such as listening to suggestions and inviting participation. Inclusive leadership reflects openness, accessibility, and availability, which can promote psychological safety (Carmeli et al., 2010). On the other hand, the leader's authority-oriented values may still communicate hierarchy, distance, and obedience. High power distance orientation reflects a preference for managerial authority and employees' acceptance of management decisions (Dorfman and Howell, 1988; Wang and Guan, 2018). This inconsistency may make employees uncertain about whether it is truly safe to express different opinions, question existing practices, or challenge the leader's views. This logic is also consistent with research showing that employees' power distance can weaken the relationship between inclusive leadership and psychological safety (Ye et al., 2019).

As a result, the positive effect of inclusive leadership on team psychological safety may become weaker.

Therefore, leader power distance orientation is expected to weaken the positive relationship between inclusive leadership and team psychological safety.

H4: Leader power distance orientation negatively moderates the relationship between inclusive leadership and team psychological safety, such that the positive relationship is weaker when leader power distance orientation is higher.

**Figure 1:**
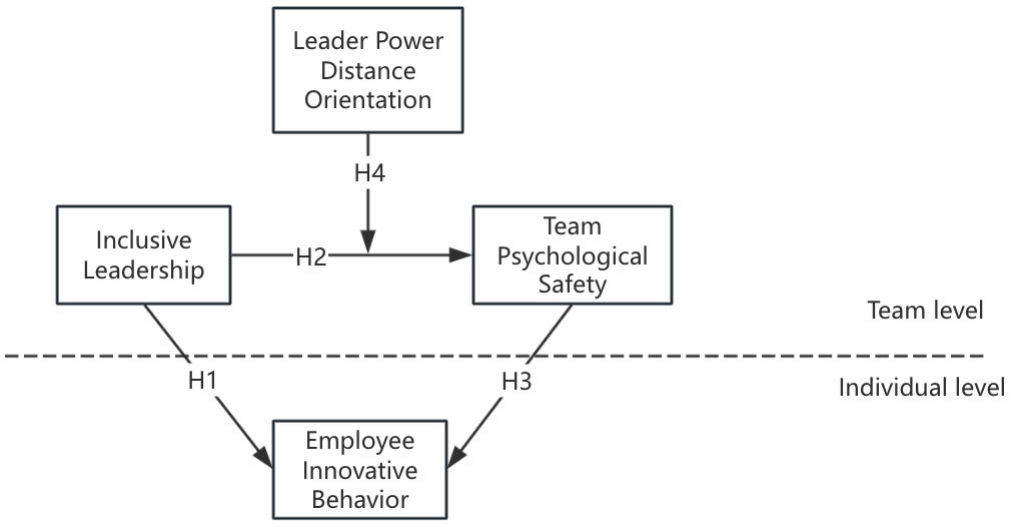
Hypothesized model.

To illustrate the proposed relationships, Figure 1 presents the hypothesized research model.

## 3 Materials and methods

### 3.1 Sample and procedure

Survey data were collected from several information technology (IT) companies in Shanghai, Beijing, Shenzhen, and Zhejiang, China. We first contacted the human resource (HR) departments of these companies and invited them to support the survey. After obtaining permission from the companies and team leaders, HR staff helped distribute the survey links to employees and team leaders. All questionnaires were administered in Chinese. To ensure translation accuracy, the questionnaire was translated and checked through a back-translation process based on Brislin's (1980) method. Before answering the survey, participants received a brief explanation of the study purpose. They were informed that participation was voluntary, that their responses would be kept confidential and used only for research purposes, and that they could withdraw at any time.

To reduce common method bias, the data were collected in three waves (Podsakoff et al., 2003). The first survey (T1) was conducted in April 2024. Employees rated inclusive leadership and provided demographic information. A total of 580 questionnaires were distributed. The second survey (T2) was conducted in May 2024. Employees completed the team psychological safety scale, and 534 valid responses were obtained. The third survey (T3) was conducted in June 2024. Team leaders rated employees' innovative behavior and reported their own leader power distance orientation. After matching the three waves of data and removing incomplete or unmatched responses, 475 employee-leader matched responses were retained.

To match the data across the three waves while maintaining anonymity, participants generated a unique identification code in each questionnaire. Only responses that could be matched across all three waves were included in the final sample. The final sample consisted of 475 employees nested within 93 teams led by 93 team leaders. Each leader rated an average of 5.11 employees, with team size ranging from 4 to 9 employees. The final matching rate was approximately 82%. Data screening and descriptive analyses were conducted using SPSS 26.0.

Among the 475 employees, 298 were male (62.74%) and 177 were female (37.26%). In terms of age, 31 employees were aged 20–29 years (6.53%), 127 were aged 30–39 years (26.74%), 201 were aged 40–49 years (42.32%), and 116 were aged 50 years or above (24.42%). Regarding education, 59 employees had an associate degree or below (12.42%), 348 held a bachelor's degree (73.26%), and 68 held a master's degree or above (14.32%). In terms of work experience, 45 employees had less than 5 years of experience (9.47%), 265 had 5–10 years of experience (55.79%), and 165 had more than 10 years of experience (34.74%).

Among the 93 team leaders, 67 were male (72.04%) and 26 were female (27.96%). In terms of age, 5 leaders were aged 20–29 years (5.38%), 18 were aged 30–39 years (19.35%), 41 were aged 40–49 years (44.09%), and 29 were aged 50 years or above (31.18%). Regarding education, 29 leaders had an associate degree or below (31.18%), 51 held a bachelor's degree (54.84%), and 13 held a master's degree or above (13.98%). In terms of work experience, 10 leaders had less than 5 years of experience (10.75%), 47 had 5–10 years of experience (50.54%), and 36 had more than 10 years of experience (38.71%).

### 3.2 Measures

Unless otherwise indicated, all items were rated on a five-point Likert scale ranging from 1 (“strongly disagree”) to 5 (“strongly agree”). The original English scales were translated into Chinese and reviewed by bilingual researchers to ensure semantic equivalence.

#### 3.2.1 Inclusive leadership

Inclusive leadership was assessed using the nine-item scale developed by (Carmeli et al. 2010). Employees evaluated their leaders' inclusive behaviors. A sample item is: “My leader is open to hearing new ideas.” Cronbach's alpha for this scale was 0.92.

#### 3.2.2 Employee innovative behavior

Employee innovative behavior was measured using the five-item scale developed by (Scott and Bruce 1994). Team leaders rated the innovative behavior of their employees. A sample item is: “This employee searches out new technologies, processes, techniques, or ideas.” Cronbach's alpha for this scale was 0.90.

#### 3.2.3 Team psychological safety

Team psychological safety was assessed using the seven-item scale developed by (Edmondson 1999). Employees reported their perceptions of psychological safety within their teams. Individual responses were aggregated to the team level for analysis. A sample item is: “In this team, it is safe to take a risk.” Cronbach's alpha for this scale was 0.94.

#### 3.2.4 Leader power distance orientation

Leader power distance orientation was assessed with the six-item scale developed by (Dorfman and Howell 1988). Team leaders reported their own power distance orientation. A sample item is: “Team members should not question their leader's decisions.” Cronbach's alpha for this scale was 0.95.

#### 3.2.5 Control variables

We controlled for demographic and organizational characteristics that may influence employee innovative behavior and team processes. At the individual level, employee gender, age, education, and work experience were included as control variables. At the team/leader level, leader gender, leader age, leader education, leader work experience, department type, company size, and team size were included as controls. Categorical control variables were dummy-coded before being entered into the multilevel models.

### 3.3 Analytical strategy

Because the data had a nested structure, with employees nested within teams, multilevel modeling was used to test the hypotheses. The main analyses were conducted in Mplus 8.3. Descriptive statistics, reliability analyses, and correlation analyses were conducted using SPSS 26.0.

Before testing the hypotheses, we first examined the measurement model through confirmatory factor analysis. We then assessed whether the employee-rated team-level variables could be aggregated to the team level. Specifically, Rwg(j), ICC(1), and ICC(2) were calculated for inclusive leadership and team psychological safety. Rwg(j) was used to evaluate within-team agreement, ICC(1) was used to assess between-team variance, and ICC(2) was used to assess the reliability of team-level means. In addition, one-way ANOVA was conducted to examine whether there were significant between-team differences.

Inclusive leadership and team psychological safety were rated by employees and were aggregated to the team level after the aggregation evidence was confirmed. Leader power distance orientation was reported by team leaders and was treated as a team-level leader characteristic. Employee innovative behavior was rated by team leaders and was analyzed as an individual-level outcome.

Employee-level control variables, including employee gender, age, education, and work experience, were specified at the within level. Leader- and team-level control variables, including leader gender, leader age, leader education, leader work experience, department type, company size, and team size, were specified at the between level. Categorical control variables were dummy-coded before being entered into the multilevel models.

Inclusive leadership and leader power distance orientation were grand mean centered before creating the interaction term. The interaction term between inclusive leadership and leader power distance orientation was entered at the between level to test the moderating effect. To examine the moderated mediation effect, conditional indirect effects were estimated at low (−1 SD) and high (+1 SD) levels of leader power distance orientation. The index of moderated mediation and its 95% confidence interval were also calculated.

### 3.4 Ethics statement

Ethical approval was not required for the studies involving humans because the research involved only anonymous survey data and no personally identifiable information was collected. The studies were conducted in accordance with local legislation and institutional requirements. Before participating in the survey, all participants were informed of the study purpose, the voluntary nature of participation, confidentiality of responses, the anonymous matching procedure, and their right to withdraw at any time. The participants provided electronic informed consent to participate in this study.

## 4 Results

### 4.1 Measurement model, reliability, and validity

To examine whether the four focal constructs were empirically distinct, confirmatory factor analysis was conducted using Mplus 8.3. As shown in Table 1, the proposed four-factor model, including inclusive leadership, team psychological safety, leader power distance orientation, and employee innovative behavior, showed good model fit: χ^2^/df = 1.801, CFI = 0.969, TLI = 0.972, RMSEA = 0.041, and SRMR = 0.032. Several alternative models were also tested. The three-factor, two-factor, and one-factor models all showed poorer fit than the proposed four-factor model. These results support the discriminant validity of the four constructs.

**Table 1:** Model fit and comparison of alternative factor structures.

Model fit index	CMIN	DF	CMIN/DF	RMSEA	TLI	CFI	SRMR
4-factor model (IL, TPS, LPDO, EIB)	572.722	318	1.801	0.041	0.972	0.969	0.032
3-factor model (IL + TPS, LPDO, EIB)	2446.787	321	7.622	0.118	0.768	0.747	0.131
2-factor model (IL + TPS + LPDO, EIB)	5178.156	323	16.031	0.178	0.471	0.425	0.234
1-factor model (IL + TPS + LPDO + EIB)	6481.104	324	20.003	0.200	0.329	0.273	0.244

IL, inclusive leadership; TPS, team psychological safety; LPDO, leader power distance orientation; EIB, employee innovative behavior.

Reliability and convergent validity were then examined. As shown in Table 2, all Cronbach's alpha values were above 0.70, indicating acceptable internal consistency. The composite reliability values were also above 0.70, and the average variance extracted values were above 0.50. These results indicate acceptable reliability and convergent validity.

**Table 2:** Reliability and validity of the scales.

Items	Cronbach's alpha	Items	Factor loading	CR	AVE
Inclusive leadership	0.915	9	0.713–0.812	0.915	0.546
Team psychological safety	0.939	7	0.808–0.839	0.939	0.687
Power distance orientation	0.948	6	0.871–0.890	0.949	0.754
Employee innovation behavior	0.902	5	0.794–0.835	0.902	0.648

Harman's single-factor test was conducted to assess potential common method bias. The largest factor accounted for 32.671% of the total variance, which was below the commonly used 50% criterion. This result suggests that common method bias was unlikely to seriously affect the findings. Nevertheless, because some variables were measured using survey responses, this issue is acknowledged in the limitations section.

### 4.2 Aggregation tests and descriptive statistics

Before testing the hypotheses, we examined whether the employee-rated variables could be aggregated to the team level. Inclusive leadership and team psychological safety were rated by employees. Leader power distance orientation was reported by team leaders and was treated as a team-level leader characteristic. Therefore, aggregation evidence was required for inclusive leadership and team psychological safety, but not for leader power distance orientation.

Following previous multilevel research, within-team agreement and between-team variance were examined using Rwg(j), ICC(1), ICC(2), and one-way ANOVA. As shown in Table 3, inclusive leadership showed acceptable aggregation evidence, with a mean Rwg(j) of 0.914, a median Rwg(j) of 0.934, ICC(1) of 0.571, and ICC(2) of 0.872. The ANOVA result was also significant, *F* = 7.805, *p* < 0.001. Team psychological safety also showed sufficient aggregation evidence, with a mean Rwg(j) of 0.904, a median Rwg(j) of 0.940, ICC(1) of 0.788, and ICC(2) of 0.950. The ANOVA result was significant, *F* = 19.995, *p* < 0.001. These results supported the aggregation of inclusive leadership and team psychological safety to the team level.

**Table 3:** Aggregation statistics.

Construct	Mean Rwg(j)	Median Rwg(j)	Range Rwg(j)	*F*	ICC(1)	ICC(2)
Inclusive leadership	0.914	0.934	0.669–0.986	7.805^***^	0.571	0.872
Team psychological safety	0.904	0.940	0.508–0.983	19.995^***^	0.788	0.950

^***^p < 0.001.

Table 4 presents the means, standard deviations, and correlations among the focal variables. Inclusive leadership was positively correlated with team psychological safety and employee innovative behavior. Team psychological safety was also positively correlated with employee innovative behavior. Leader power distance orientation was negatively correlated with inclusive leadership, team psychological safety, and employee innovative behavior. Employee innovative behavior was measured at the individual level and was averaged at the team level only for descriptive and correlation analyses.

**Table 4:** Descriptive statistics and correlations.

Variable	M	SD	1	2	3	4
1. EIB	3.297	0.952	1			
2. IL	3.351	0.714	0.485^***^	1		
3. TPS	3.203	1.032	0.530^***^	0.434^***^	1	
4. LPDO	3.167	0.881	−0.261^*^	−0.279^**^	−0.401^***^	1

*N* = 93 teams. EIB, employee innovative behavior; IL, inclusive leadership; TPS, team psychological safety; LPDO, leader power distance orientation. EIB was averaged at the team level for descriptive and correlation analyses only. ^*^p < 0.05, ^**^p < 0.01, ^***^p < 0.001.

### 4.3 Multilevel hypothesis testing

After confirming the suitability of aggregation, the hypotheses were tested using multilevel path analysis. Employee-level control variables were specified at the within level, whereas leader- and team-level control variables were specified at the between level. The results are reported in Table 5.

**Table 5:** Multilevel path analysis results.

Outcome	Predictor	*B*	SE	*z*	*p*	95% CI
EIB	IL	0.473	0.150	3.160	0.002	[0.180, 0.767]
EIB	TPS	0.357	0.097	3.693	<0.001	[0.167, 0.546]
TPS	IL	0.473	0.126	3.761	<0.001	[0.227, 0.719]
TPS	LPDO	−0.420	0.117	−3.602	<0.001	[−0.649, −0.192]
TPS	IL × LPDO	−0.341	0.128	−2.659	0.008	[−0.592, −0.090]
Indirect effect	IL → TPS → EIB	0.169	0.066	2.574	0.010	[0.040, 0.297]

Unstandardized estimates are reported. Employee-level controls were included at the within level; leader/team-level controls were included at the between level. *R*^2^ = 0.362 for TPS and 0.439 for between-level EIB. Model fit: χ^2^(2) = 0.363, *p* = 0.834, RMSEA = 0.000, CFI = 1.000, SRMR within = 0.000, SRMR between = 0.007.

Inclusive leadership had a significant positive effect on employee innovative behavior (B = 0.473, SE = 0.150, *z* = 3.160, *p* = 0.002, 95% CI [0.180, 0.767]), supporting Hypothesis 1. Inclusive leadership also had a significant positive effect on team psychological safety (B = 0.473, SE = 0.126, *z* = 3.761, *p* < 0.001, 95% CI [0.227, 0.719]), supporting Hypothesis 2.

Team psychological safety had a significant positive effect on employee innovative behavior (B = 0.357, SE = 0.097, *z* = 3.693, *p* < 0.001, 95% CI [0.167, 0.546]). Thus, Hypothesis 3 was supported. The indirect effect of inclusive leadership on employee innovative behavior through team psychological safety was also significant (B = 0.169, SE = 0.066, *z* = 2.574, *p* = 0.010, 95% CI [0.040, 0.297]), indicating that team psychological safety served as a mediating mechanism.

### 4.4 Moderation and moderated mediation

Hypothesis 4 predicted that leader power distance orientation would weaken the positive relationship between inclusive leadership and team psychological safety. As shown in Table 5, the interaction between inclusive leadership and leader power distance orientation was negative and significant (B = −0.341, SE = 0.128, *z* = −2.659, *p* = 0.008, 95% CI [−0.592, −0.090]). This result indicates that the positive effect of inclusive leadership on team psychological safety became weaker when leader power distance orientation was higher. Thus, Hypothesis 4 was supported.

To further interpret the interaction effect, a simple slope analysis was conducted. As shown in Table 6, when leader power distance orientation was low, inclusive leadership had a significant positive effect on team psychological safety (B = 0.773, SE = 0.152, *z* = 5.083, *p* < 0.001, 95% CI [0.475, 1.071]). However, when leader power distance orientation was high, this effect was not significant (B = 0.173, SE = 0.184, *z* = 0.939, *p* = 0.348, 95% CI [−0.188, 0.534]). The difference between the two slopes was significant (B = 0.600, SE = 0.226, *z* = 2.659, *p* = 0.008, 95% CI [0.158, 1.042]).

**Table 6:** Simple slope analysis.

LPDO level	Effect of IL on TPS	SE	*z*	*p*	95% CI
Low LPDO (−1 SD)	0.773	0.152	5.083	<0.001	[0.475, 1.071]
High LPDO (+1 SD)	0.173	0.184	0.939	0.348	[−0.188, 0.534]
Difference	0.600	0.226	2.659	0.008	[0.158, 1.042]

**Figure 2:**
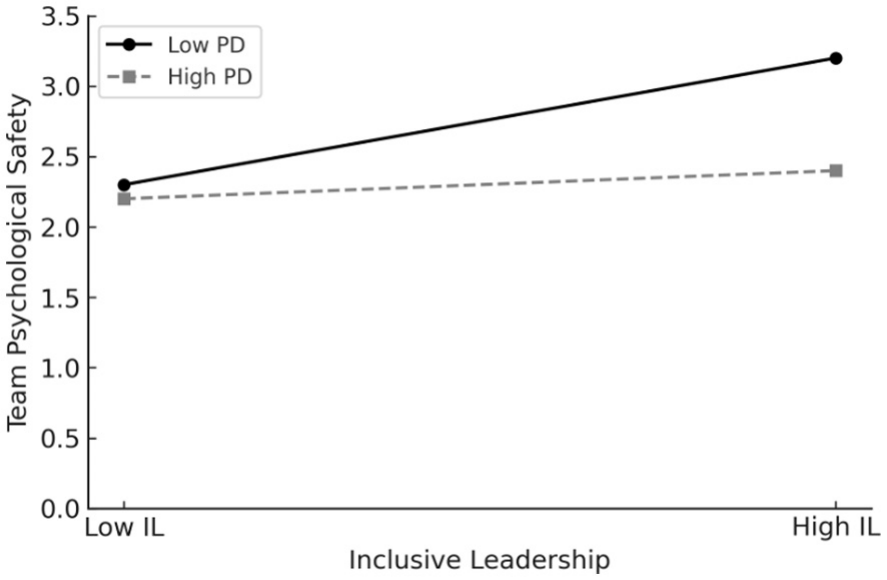
Moderating role of leader power distance orientation in the inclusive leadership-team psychological safety relationship.

Figure 2 presents the interaction effect of inclusive leadership and leader power distance orientation on team psychological safety.

Finally, the moderated mediation effect was examined. As shown in Table 7, the conditional indirect effect of inclusive leadership on employee innovative behavior through team psychological safety was significant when leader power distance orientation was low (B = 0.276, SE = 0.086, *z* = 3.205, *p* = 0.001, 95% CI [0.107, 0.444]), but not significant when leader power distance orientation was high (B = 0.062, SE = 0.071, *z* = 0.874, *p* = 0.382, 95% CI [−0.077, 0.200]). The index of moderated mediation was significant and negative (B = −0.121, SE = 0.049, *z* = −2.454, *p* = 0.014, 95% CI [−0.218, −0.024]). These results indicate that leader power distance orientation weakened the indirect effect of inclusive leadership on employee innovative behavior through team psychological safety.

**Table 7:** Conditional indirect effects and index of moderated mediation.

Condition	Effect	SE	*z*	*p*	95% CI
Low LPDO (−1 SD)	0.276	0.086	3.205	0.001	[0.107, 0.444]
High LPDO (+1 SD)	0.062	0.071	0.874	0.382	[−0.077, 0.200]
Index of moderated mediation	−0.121	0.049	−2.454	0.014	[−0.218, −0.024]

LPDO, leader power distance orientation. Unstandardized estimates are reported.

## 5 Discussion

This study examined how and when inclusive leadership promotes employee innovative behavior. The results showed that inclusive leadership was positively related to employee innovative behavior and team psychological safety. Team psychological safety further promoted employee innovative behavior and mediated the relationship between inclusive leadership and employee innovative behavior. In addition, leader power distance orientation weakened the positive relationship between inclusive leadership and team psychological safety. The conditional indirect effect of inclusive leadership on employee innovative behavior through team psychological safety was significant when leader power distance orientation was low, but not significant when leader power distance orientation was high. These findings suggest that inclusive leadership promotes innovation through a psychologically safe team climate, but this process depends on leaders' power distance orientation.

### 5.1 Theoretical implications

This study develops and tests an integrated multilevel framework to explain how inclusive leadership influences employee innovative behavior and under what conditions this effect becomes stronger or weaker. This study makes several theoretical contributions.

First, this study advances the theoretical understanding of how inclusive leadership relates to employee innovative behavior. Prior research has shown that inclusive leadership can promote employee creativity and innovative behavior (Fang et al., 2019). However, existing studies have often explained this relationship through individual-level psychological mechanisms, such as psychological capital, perceived organizational support, positive emotions, and creative self-efficacy (Qi et al., 2019; He and Li, 2026). This study extends this line of research by identifying team psychological safety as a team-level mechanism. Team psychological safety refers to a shared belief among team members that expressing ideas, proposing new suggestions, and taking interpersonal risks are safe within the team (Edmondson, 1999). The results show that inclusive leadership can foster team psychological safety by creating a supportive team environment, which in turn stimulates employees' innovative behavior. Thus, this study shows that inclusive leadership promotes innovation not only by influencing individual employees, but also by shaping a shared team climate.

Second, this study deepens the understanding of how leadership behaviors influence employee outcomes from a multilevel perspective. Organizations are multilevel systems, and leadership behaviors often operate at the team level while influencing individual outcomes through shared team perceptions and interaction patterns among team members (Klein and Kozlowski, 2000). However, in the inclusive leadership literature, many studies still rely on single-level analyses and pay limited attention to the cross-level processes through which leadership behaviors shape employee outcomes via team environments. (Li and Tang, 2022) also noted that inclusive leadership has rarely been examined at both individual and team levels. By employing multilevel analysis, this study links team-level inclusive leadership and team psychological safety with individual-level employee innovative behavior. In this way, the study provides evidence for a cross-level process in which leadership behavior shapes a team climate, and this team climate further influences individual innovative behavior.

Third, this study identifies leader power distance orientation as an important boundary condition of inclusive leadership. Prior research has shown that inclusive leadership is generally related to positive employee and team outcomes (Veli Korkmaz et al., 2022). However, the effectiveness of leadership behaviors may depend on whether employees see these behaviors as consistent and credible. This idea is consistent with behavioral integrity theory, which emphasizes the perceived alignment between a manager's words, actions, and values (Simons, 2002). The results show that leader power distance orientation weakens the positive relationship between inclusive leadership and team psychological safety. This finding suggests that inclusive leadership is more effective when leaders' inclusive behaviors are consistent with values of equality and participation. In this way, this study explains why inclusive leadership may build psychological safety more strongly in some teams than in others.

In contrast, when leaders have high power distance orientation, they may still emphasize authority, hierarchy, and distance (Kwan et al., 2025). Even if they show inclusive behaviors, employees may receive mixed signals. They may feel uncertain about whether speaking up and taking interpersonal risks are truly safe (Edmondson, 1999).

As a result, the positive effect of inclusive leadership on team psychological safety becomes weaker. More importantly, the moderated mediation results show that leader power distance orientation weakens not only the first-stage relationship between inclusive leadership and team psychological safety, but also the overall indirect process through which inclusive leadership promotes employee innovative behavior. By revealing this process, this study highlights the important role of leaders' cultural values in shaping the effectiveness of inclusive leadership. It also suggests that the effect of inclusive leadership depends not only on leaders' visible actions, but also on whether these actions are consistent with their underlying values.

### 5.2 Practical implications

First, organizations should encourage leaders to use inclusive leadership behaviors. Inclusive leaders listen to employees' ideas, respect different opinions, and encourage employees to take part in decision making. These behaviors help create an open and supportive work environment. When employees feel that their opinions are respected, they are more willing to share new ideas and try new ways of doing their work. Therefore, organizations should include inclusive leadership in leadership training and management development programs.

Second, managers should try to build team psychological safety. A safe team environment allows employees to speak freely, ask questions, and share their ideas without fear. Leaders can build such an environment by encouraging open communication, accepting mistakes as part of learning, and giving helpful feedback. When employees feel safe in the team, they are more willing to share creative ideas and explore new solutions.

Third, organizations should pay attention to leaders' power distance orientation when they apply inclusive leadership practices. This study shows that inclusive leadership may be less effective when leaders hold strong authority-oriented values. In this case, employees may receive mixed signals. They may hear that participation is encouraged, but they may still feel that the leader values hierarchy and control. Therefore, leadership training should not only teach inclusive behaviors but also help leaders reflect on their assumptions about authority and participation. Leaders with high power distance orientation may need to build trust gradually, invite employee input more consistently, and give clear responses to employee suggestions. In this way, inclusive leadership behaviors can become more credible and effective.

### 5.3 Limitations and future research

First, the sample was collected from IT firms in China. Although the IT industry provides a suitable context for studying innovation, the findings may not fully generalize to other industries or cultural settings. In addition, a relatively large proportion of employees in the sample were in their 40s. This may reflect the inclusion of experienced employees, project members, and middle-level staff, but future studies could use more diverse samples to examine whether the findings hold across different age groups, industries, and cultural contexts.

Second, although the data were collected in three waves, the research design cannot fully establish causal relationships. Future studies may use longitudinal or experimental methods to more clearly test the causal links among inclusive leadership, team psychological safety, and employee innovative behavior. Such designs would help explain how inclusive leadership behaviors gradually shape team psychological safety over time.

Third, although this study used a three-wave and multi-source design, common method bias cannot be fully ruled out. Inclusive leadership and team psychological safety were both rated by employees, which may create some shared method variance. Future studies could collect data from more sources or use objective indicators of innovation to reduce this concern.

Fourth, this study focused on team psychological safety as the core team-level mechanism. Future research could further examine how psychological safety develops over time in response to repeated inclusive leadership behaviors. This would help explain the dynamic process through which leader behaviors become shared team climate.

Finally, this study examined leader power distance orientation as a boundary condition related to leader values. Future research could further explore the consistency between leaders' visible behaviors and underlying values. For example, future studies may examine whether employees' perceived leader behavioral integrity explains why high leader power distance orientation weakens the effect of inclusive leadership.

## Data Availability

The anonymized data supporting the conclusions of this article are available from the corresponding author upon reasonable request.
